# A combination analysis based on bioinformatics tools reveals new signature genes related to maternal obesity and fetal programming

**DOI:** 10.3389/fmed.2024.1434105

**Published:** 2024-09-04

**Authors:** Chunhong Liu, Yulan Lu, Chunchuan Huang, Yonglong Zeng, Yuye Zheng, Chunfang Wang, Huatuo Huang

**Affiliations:** ^1^Center for Medical Laboratory Science, The Affiliated Hospital of Youjiang Medical University for Nationalities, Baise, China; ^2^Baise Key Laboratory for Research and Development on Clinical Molecular Diagnosis for High-Incidence Diseases, Baise, China; ^3^Key Laboratory of Research on Clinical Molecular Diagnosis for High Incidence Diseases in Western Guangxi, Baise, China; ^4^Department of Medical Reproduction Center, The Affiliated Hospital of Youjiang Medical University for Nationalities, Baise, China; ^5^Department of Rehabilitation Medicine, The Traditional Chinese Medicine Hospital of Baise City, Basie, China

**Keywords:** pregnancy, obesity, placenta, bioinformatics, fetal programming

## Abstract

**Background:**

Maternal obesity significantly influences fetal development and health later in life; however, the molecular mechanisms behind it remain unclear. This study aims to investigate signature genes related to maternal obesity and fetal programming based on a genomic-wide transcriptional placental study using a combination of different bioinformatics tools.

**Methods:**

The dataset (GSE128381) was obtained from Gene Expression Omnibus (GEO). The data of 100 normal body mass index (BMI) and 27 obese mothers were included in the analysis. Differentially expressed genes (DEGs) were evaluated by limma package. Thereafter, functional enrichment analysis was implemented. Then, weighted gene co-expression network analysis (WGCNA) and the least absolute shrinkage and selection operator (LASSO) analysis were used to further screening of signature genes. Simple linear regression analysis was used to assess the correlation between signature genes and newborn birth weight. Gene set enrichment analysis (GSEA) was implemented to study signaling pathways related to signature genes. The expression of the signature genes was also explored in 48 overweight mothers in the same dataset.

**Results:**

A total of 167 DEGs were obtained, of which 122 were up-regulated while 45 were down-regulated. The dataset was then clustered into 11 modules by WGCNA, and the MEbrown was found as the most significant module related to maternal obesity and fetal programming (cor = 0.2, *p* = 0.03). The LASSO analysis showed that *PTX3*, *NCF2*, *HOXB5*, *ABCA6*, and *C1orf162* are signature genes related to maternal obesity and fetal programming, which were increased in the placenta of obese mothers compared to those with normal BMI. The area under the curve (AUC) of the signature genes in the receiver operating characteristic curve (ROC) was 0.709, 0.660, 0.674, 0.667, and 0.717, respectively. Simple linear regression analysis showed that HOXB5 was associated with newborn birth weight. GSEA analysis revealed that these signature genes positively participate in various signaling pathways/functions in the placenta.

**Conclusion:**

*PTX3*, *NCF2*, *HOXB5*, *ABCA6*, and *C1orf162* are novel signature genes related to maternal obesity and fetal programming, of which *HOXB5* is implicated in newborn birth weight.

## Introduction

Obesity has increased as a worldwide public health concern during the past decades (1). The obese population was reported to increase from 1990 to 2022 in both men and women in a previous study that included 200 countries and territories (81% for women and 70% for men). Similarly, it was reported that 39% of adults were overweight, of which 13% of the population was obese in 2016 in the world (1). Nowadays, obesity in women of reproductive age has been a common clinical condition. For example, those women of reproductive age with obesity increased from 28.4 to 34.0% from 1999 to 2010 in America (2). Moreover, the global age-standardized mean body mass index (BMI) increased from 22.1 in 1975 to 24.4 in 2014, and the prevalence of obesity doubled between 1980 and 2008 in women (3). It is well-established that maternal obesity generates an obesogenic environment that has long-term effects on fetal development and health later in life (4, 5). Those fetuses from obese mothers have a significantly higher risk for adverse clinical outcomes and even death, and they also present a higher risk of developing cardiovascular diseases and metabolic disorders later in life (6, 7). However, the molecular mechanisms involved remain unknown.

The placenta is the primary regulating organ of the intrauterine environment, which is critically important for the development and health of a fetus (8). Functionally, the placenta plays an important role in transporting nutrients to the growing fetus. The placenta is also known as a powerful endocrine organ for hormone secretion in physiological adaption during pregnancy (9). The placenta works like a shield to protect the fetus from detrimental environments such as toxic substances and microbes (10). However, when the environmental stresses have exceeded the capacity of the placenta, the intrauterine environment may be disrupted, which may have long-lasting consequences for fetus development and health later in life.

Regardless of the vital role of the placenta in fetal development, few studies were performed on the genome-wide influence of maternal obesity on the placental transcriptome. A recent study reported by Cox et al. has drawn a lot of public attention by offering valuable insight into the knowledge about the relation between maternal prepregnant BMI and newborn birth weight based on human placental samples (10). In this study, the authors employed weighted gene co-expression network analysis (WGCNA) to study the correlation between maternal BMI [including BMI and BMI categories (underweight, overweight, obese)] and newborn birth weight [including birth weight and birth weight categories (low and high birth weight)] (10). However, their major findings were based on maternal BMI, but not other BMI categories, such as underweight, overweight, or obesity, that were associated with newborn birth weight (10). Moreover, their final hub genes were based on the functional enrichment analysis, which may be interesting by using other well-established bioinformatic predicting methods such as least absolute shrinkage and selection operator (LASSO) analysis and random forest (10). A bioinformatics analysis study based primarily on the obesity population to clarify its effect on fetal programming via the placenta as intermedia will serve as a supplement to this interesting original study. Therefore, this study aims to use a combination of multiple bioinformatic tools, including the limma package for screening differentially expressed genes (DEGs) together with WGCNA for critical co-expression module as well as LASSO to explore signature genes based on the dataset from Cox et al. (10). Moreover, gene set enrichment analysis (GSEA) was performed to better understand the role of these signature genes in the pathophysiology that occurs in obese pregnancy and their potential role in fetus development.

## Materials and methods

### Data sources

**FIGURE 1:**
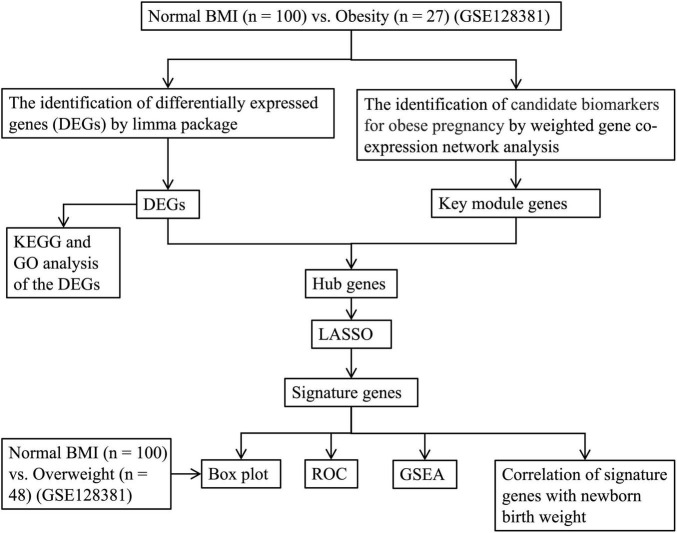
The flow chart of the study.

The dataset GSE128381 (Platform: GPL17077; Microarray 039381) was downloaded from GEO.^1^ Our study included data from placentas of 100 mothers with normal prepregnant BMI and 27 mothers with prepregnant obesity (BMI > 30). The details of clinical data of the populations can be seen in Supplementary Table 1. The flow chart of the study is shown in Figure 1.

### Identification of DEGs

The DEGs between mothers with obesity and normal pregnancy were screened using limma package (11), with criteria as listed below: p-value less than 0.05 and log | Fold change (FC)| larger than 0.3. The results of DEGs were generated and displayed by volcano plot. Additionally, the top 50 up-regulated and 50 down-regulated DEGs were shown by heatmap.

### Functional and pathway enrichment analyses

The functional enrichment analyses of DEGs were evaluated by the clusterProfiler package, on account of Gene Ontology (GO) and Kyoto Encyclopedia of Genes and Genomes (KEGG) (12). Three categories including biological process (BP), cellular component (CC), and molecular function (MF), were used to explore the biological functions of the identified DEGs. Signaling pathways involved in maternal obesity and fetal programming were studied using KEGG analysis.

### WGCNA and LASSO analysis

The co-expression network in GSE128381 was constructed by WGCNA based on the scale-free topology criterion (13). The soft threshold power and adjacencies were calculated using the pickSoftThreshold function of the WGCNA package. The adjacency matrix was transformed into a topological overlap matrix. Additionally, the corresponding dissimilarity was calculated for a hierarchical clustering analysis. The co-expressed gene modules of this study were screened through dynamic tree-cutting. The module with the highest correlation coefficient (cor) and lowest p-value was selected as the key module. The hub genes of the current study were identified through intersecting DGEs and genes in the screened key module from WGCNA. Thereafter a machine learning algorithm, the LASSO, which was performed by the glmnet R package (14), was used to screen out signature genes further.

### The expression and diagnostic performance of the signature genes

The expression of the signature genes was exhibited by box plots. The AUC from the ROC was used to evaluate the representativeness of identified signature genes with maternal obesity and fetal programming. An AUC larger than 0.65 was used to class diagnostic performance.

### The expression and diagnostic performance of the signature genes in the overweight samples

We also explored the expression and the diagnostic performance of the signature genes by the overweight data in the same dataset. This comparison includes 100 placenta samples from normal BMI mothers and 48 placenta samples from prepregnant overweight mothers. Box plot and AUC of ROC were used for the validation.

### Simple linear regression analysis

Simple linear regression analysis was applied to explore the association between the expression of signature genes and newborn birth weight.

### GSEA analysis

We further performed the GSEA analysis of the signature genes based on the KEGG MEDICUS database, which includes information regarding KEGG PATHWAY, NETWORK, DISEASE, and DRUG databases, as well as FDA drug labels by the GSEA software (V4.3.2) and included relations that have a p-value less than 0.05 (15).

### Statistical analysis

Statistical analyses in our current study were carried out using R software (Version 4.3.1) and GraphPad Prism (Version 9.5.0). A p-value less than 0.05 was used as statistically significant.

## Results

### Identification of DEGs between mothers with obesity and normal pregnancy

**FIGURE 2:**
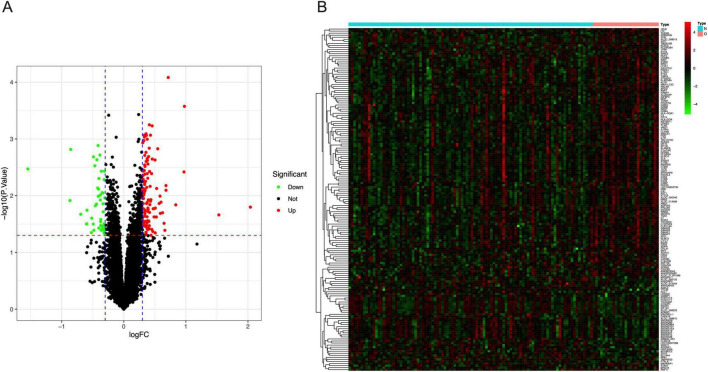
DEGs between normal BMI and obese mothers. **(A)** The volcano plot showed the fold change of DEGs in obese mothers compared to those with normal BMI. **(B)** The heatmap of the top 50 up-regulated and 45 down-regulated DEGs in the placenta of obese mothers.

167 DEGs were identified, of which 122 of them were up-regulated while 45 were down-regulated (Figure 2A). The heatmap demonstrated the top 50 up-regulated and 45 down-regulated DEGs between mothers with obesity and normal pregnancy (Figure 2B).

### Function enrichment analysis of the DEGs

**FIGURE 3:**
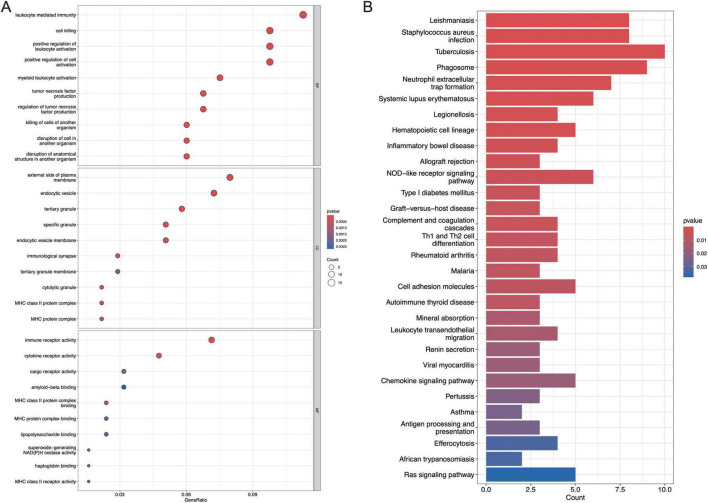
Functional enrichment analysis of DEGs. **(A)** The top 10 functional enrichment in BP, CC, and MF analysis, respectively, **(B)** the top 30 signaling pathways involved by DEGs in the KEGG analysis.

The GO analysis consists of three categories, including BP, CC, and MF. As is shown in Figure 3A, the top three enriched pathways for BP analysis are leukocyte-mediated immunity, cell killing, and positive regulation of leukocyte activation, respectively. In the CC analysis, external side of plasma membrane, endocytic vesicle, and tertiary granule appeared to be the top three locations. Additionally, immune receptor activity, cytokine receptor activity as well as cargo receptor activity played a significant role in MF. As to the KEGG analysis, the top 3 enriched pathways are leishmaniasis, staphylococcus aureus infection, and tuberculosis (Figure 3B).

### Signature genes identified by WGCNA and LASSO

**FIGURE 4:**
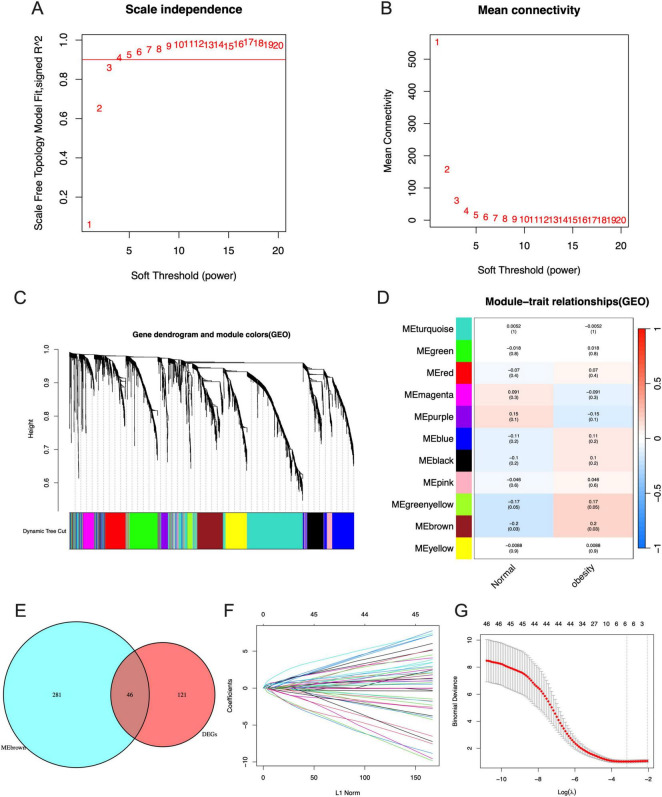
Identification of signature genes by WGCNA and LASSO analysis. **(A)** The scale independence of WGCNA. **(B)** The mean connectivity of WGCNA. **(C)** The cluster dendrogram of WGCNA. **(D)** The clustered modules of WGCNA. **(E)** The interaction between DEGs and genes in MEbrown module by Venn diagram. **(F)** Penalty plot of the LASSO model with error bars denoting standard errors. **(G)** The LASSO coefficients for signature genes.

In the WGCNA, the numbers in Figures 4A, B were the beta values that were used to determine the best power for the scale free topology model fit index and mean connectivity to get the Person Coefficient between genes, which is important to identify co-expression genes to construct the following gene dendrogram. We can see that the optimal soft threshold power was determined at 3 with a fit index of 0.86 (Figures 4A, B). The cluster dendrogram of the analysis is shown in Figure 4C. Ultimately, the data was clustered into 11 modules (Figure 4D). The association between each module and maternal obesity and fetal programming was calculated. The results showed that the MEbrown module was significantly correlated to maternal obesity and fetal programming (cor = 0.2, *p* = 0.03), which includes 327 genes. The Venn diagram was applied to analyze the overlap between genes in the MEbrown module and the DEGs, in which 46 hub genes were obtained (Figure 4E). All hub genes are listed in Table 1. Thereafter, a machine algorithm, namely LASSO, was used to screen out signature genes from hub genes further. As a result, 5 signature genes were obtained (Figures 4F, G). These signature genes include pentraxin 3 (*PTX3*), neutrophil cytosolic factor 2 (*NCF2*), homeobox protein Hox-B5 (*HOXB5*), ATP binding cassette subfamily A member 6 (*ABCA6*), and chromosome 1 open reading frame 162 (*C1orf162*).

**TABLE 1:** Hub genes from the intersect between genes from DEGs and WGCNA.

Item	Hub genes
Intersect between genes from DEGs and WGCNA	ABCA6, ALOX5AP, AMICA1, C1QB, C1orf162, C3, CD33, CD37, CD53, CD74, CYBB, FCGR3A, FGR, GZMB, GZMH, HLA-DOA, HLA-DQA1, HOXB5, IL1RL1, IL7R, INMT, IRAK3, ITGB2, LILRA6, LYVE1, MRC1, MT1E, MT1M, NCF2, NKG7, NLRC4, PLA2G4A, PROK1, PTGER2, PTX3, RNASE6, RUNX3, SLA, SLAMF8, STAB1, TBC1D10C, TCN2, TIMP4, TLR2, TXNIP, VMO1

### Relative expression of signature genes between mothers with obesity and normal BMI and their correlation with maternal obesity and fetal programming

**FIGURE 5:**
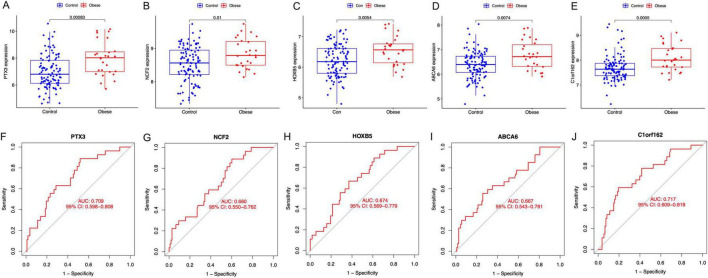
The expression and the diagnostic performance of the signature genes between normal BMI (Control) and obese mothers. **(A)** The expression of PTX3, **(B)** the expression of NCF2, **(C)** the expression of HOXB5, **(D)** the expression of ABCA6, **(E)** the expression of C1orf162, **(F)** the ROC curves for PTX3, **(G)** The ROC curves for NCF2, **(H)** the ROC curves for HOXB5, **(I)** the ROC curves for ABCA6, and **(J)** the ROC curves for C1orf162.

The expression of *PTX3*, *NCF2*, *HOXB5*, *ABCA6*, and *C1orf162* were upregulated in the placenta of mothers with obesity compared with mothers with normal BMI (Figures 5A–E). Moreover, the AUC of the ROC of these signature genes was 0.709 (*PTX3*), 0.660 (*NCF2*), 0.674 (*HOXB5*), and 0.667 (*ABCA6*), 0.717 (*C1orf162*), respectively (Figures 5F–J). Indicating that these signature genes have a relatively good value in representing features of maternal obesity and pathological factors affecting fetus development.

### Relative expression of signature genes between mothers with prepregnant overweight and normal BMI and their correlation with maternal overweight and fetal programming

**FIGURE 6:**
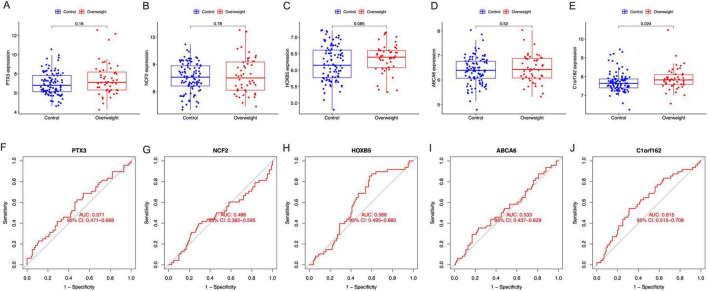
The expression and the diagnostic performance of the signature genes in normal BMI and overweight mothers. **(A)** The expression of PTX3, **(B)** the expression of NCF2, **(C)** the expression of HOXB5, **(D)** the expression of ABCA6, **(E)** the expression of C1orf162, **(F)** the ROC curves for PTX3, **(G)** the ROC curves for NCF2, **(H)** the ROC curves for HOXB5, **(I)** the ROC curves for ABCA6, and **(J)** the ROC curves for C1orf162.

The results showed that the expression of *C1orf162* was significantly increased in the placentas of mothers with prepregnant overweight compared to those with normal BMI (Figure 6E); however, no difference was observed in four other signature genes, even though an increased trend was found in the expression of *PTX3*, *HOXB5*, and *ABCA6* in mothers with overweight (Figures 6A–D). The AUC of the five signature genes were 0.571 (*PTX3*), 0.486 (*NCF2*), 0.588 (*HOXB5*), and 0.533 (*ABCA6*), 0.615 (*C1orf162*), respectively (Figures 6F–J).

### Correlation between signature genes and newborn birth weight

**FIGURE 7:**
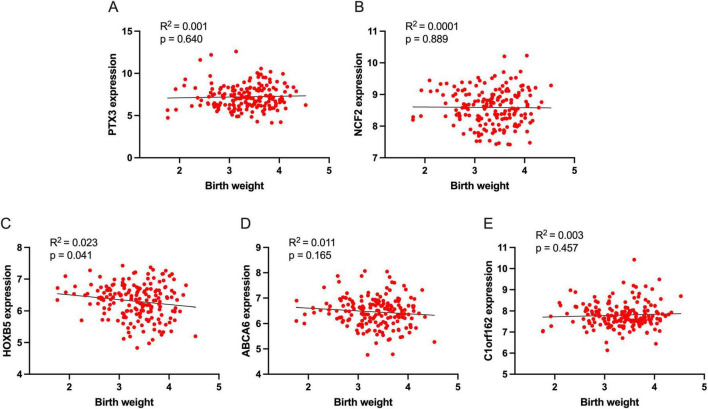
The association of signature genes with newborn birth weight. **(A)** The association of PTX3 with birth weight, **(B)** The association of NCF2 with birth weight, **(C)** The association of HOXB5 with birth weight, **(D)** The association of ABCA6 with birth weight, and **(E)** The association of C1orf162 with birth weight.

We also evaluated the correlation between signature genes and newborn birth weight, and the results are shown in Figures 7A–E. Among these signature genes, only *HOXB5* directly correlates with the newborn birth weight (Figure 7C, R^2^ = 0.023, *p* = 0.041).

### GSEA analysis

**FIGURE 8:**
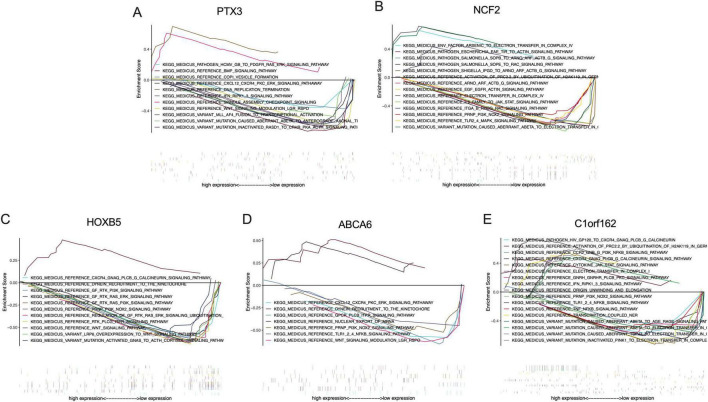
The KEGG analysis of the signature genes via GSEA. **(A)** Signaling pathways involved by PTX3 in the placenta of obese mothers, **(B)** Signaling pathways involved by NCF2 in the placenta of obese mothers, **(C)** signaling pathways involved by HOXB5 in the placenta of obese mothers, **(D)** signaling pathways involved by ABCA6 in the placenta of obese mothers, and **(E)** signaling pathways involved by C1orf162 in the placenta of obese mothers.

Up to ten signaling pathways/functions of each signature gene are shown in Figure 8. *PTX3* participates in the regulation of DNA replication termination, spindle assembly checkpoint signaling, COPI vesicle formation, CXCL12-CXCR4-PKC-ERK signaling pathway, and Wnt signaling modulation (Figure 8A). *NCF2* is associated with mutation-caused aberrant Abeta to electron transfer in Complex IV, electron transfer in Complex IV, arsenic to electron transfer in Complex IV, EGF-EGFR-Actin signaling pathway, ARNO-ARF-ACTB_G signaling pathway, PRNP-PI3K-NOX2 signaling pathway, ITGA/B-RHOG-RAC signaling pathway, TLR2/4-MAPK signaling pathway, and IL6 family to Jak-STAT signaling pathway (Figure 8B). *HOXB5* is involved in the regulation of dynein recruitment to the kinetochore, PRNP-PI3K-NOX2 signaling pathway, GF-RTK-PI3K signaling pathway, GF-RTK-RAS-PI3K signaling pathway, GF-RTK-RAS-ERK signaling pathway, Wnt signaling pathway, regulation of GF-RTK-RAS-ERK signaling, LRP6-overexpression to Wnt signaling pathway, mutation-activated GNAS to ACTH-cortisol signaling pathway, RTK-PLCG-ITPR signaling pathway, and CXCR4-GNAQ-PLCB/G-calcineurin signaling pathway (Figure 8C). *ABCA6* is associated with the regulation of dynein recruitment to the kinetochore, nuclear export of mRNA, PRNP-PI3K-NOX2 signaling pathway, CXCL12-CXCR4-PKC-ERK signaling pathway, GPCR-PLCB-ITPR signaling pathway, Wnt signaling modulation, and TLR1/2/4-NFKB signaling pathway (Figure 8D). *C1orf162* participates in mutation-inactivated PINK1 to electron transfer in Complex I, transcription-coupled NER, electron transfer in Complex I, origin unwinding and elongation, mutation-caused aberrant Abeta to electron transfer in Complex I, mutation-caused aberrant TDP43 to electron transfer in Complex I, TLR1/2/4-NFKB signaling pathway, CCR2-GNB/G-PI3K-NFKB signaling pathway, cytokine-Jak-STAT signaling pathway, CXCR4-GNAQ-PLCB/G-calcineurin signaling pathway, TNF-NFKB signaling pathway, mutation-caused aberrant Abeta to AGE-RAGE signaling pathway, and PRNP-PI3K-NOX2 signaling pathway (Figure 8E).

## Discussion

In this study, we identified a total of 167 DEGs, of which 122 were up-regulated, and 45 were down-regulated from the placenta of obese mothers. In the following bioinformatic screening, five signature genes, including *PTX3*, *NCF2*, *HOXB5*, *ABCA6*, and *C1orf162*, were identified as related to maternal obesity and fetal programming and were increased in obese mothers compared to those with normal BMI. Interestingly, we found that the *HOXB5* was associated directly with newborn birth weight. GSEA analysis revealed that these signature genes work either individually or coordinately in various signaling pathways and functions in the placenta.

The role of *PTX3* in health and diseases has been widely documented, especially in inflammation, oxidative stress, immune response, cardiovascular diseases, and metabolic diseases. *PTX3* is an acute-phase protein that is similar to the C-reactive protein in both structure and function. *PTX3* has been regarded as a favorable marker for inflammation (16). Elevated *PTX3* was reported in subjects with obesity and was correlated with a higher frequency of metabolic syndrome and more hepatic steatosis (17, 18). *PTX3* was suggested as a novel target of inflammation cascade in obesity (19). *PTX3*, therefore, may play a critical role in maternal obesity and fetal programming.

The *NCF2* protein is a cytosolic subunit of NADPH oxidase located in neutrophils. Literature reports concerning *NCF2* were mostly involved in immune infiltration, oxidative stress, and inflammation-related diseases, such as chronic granulomatous disease, major depressive disorder, and non-alcoholic fatty liver disease (20–22). Obesity is a chronic metabolic disorder characterized by systemic inflammation and systemic oxidative stress in peripheral tissues. Several transcriptomic analyses showed that *NCF2* was a favorable signature gene in obese-related complications (23–25). Patients who received bariatric surgery displayed downregulated *NCF2* expression (25). Taken together, *NCF2* may be a promising research target for obesity and may represent a good target gene for studying maternal obesity and fetal programming, even though further studies are imperative.

The *HOXB5* gene belongs to a member of the Antp homeobox family, which encodes a nuclear protein that has a homeobox DNA-binding domain. *HOXB5* functions as a sequence-specific transcription factor in cells. To date, little is known about the functions of *HOXB5* genes with obesity. A previous study showed that the expression of several homeobox genes, including *HOXB5*, increased following fat loss after bariatric surgery, which to some extent emphasizes the importance of homeobox transcription in adipose tissue (26). However, it is unclear whether the increased expression of *HOXB5* may promote fat loss or simply a response to changes in adipose tissue mass. A positive role of homeobox genes in tissue repair and regeneration has been observed in a previous study (27). *HOXB5* was suggested to play a protective role in obesity by mitigating inflammation and promoting adipocyte turnover (26). However, in our study, we found the expression of *HOXB5* was increased in the placenta of obese mothers. We hypothesize that this phenomenon may be a consequence of the response of the placenta to the stressful environment of obesity to maintain the function of the placenta. The negative correlation between *HOXB5* expression and newborn birth weight supports the protective role of the *HOXB5* protein.

*ABCA6* is a membrane-associated protein that is a member of the ATP-binding cassette (ABC) transporters superfamily. ABC proteins help to transport various molecules through extra- and intracellular membranes (28). During pregnancy, ABC transporters control the transfer of several nutrients in the placental, including steroids, drugs, chemicals, and immunological factors at the fetal-maternal interface (29). Most of the previous reports of *ABCA6* were on cancers. However, the role of *ABCA6* in obesity has never been reported before. A previous report showed that the expression of *ABCA6* protein changed following trimesters and was significantly higher in the third trimester compared to the first trimester (29). Our results confirmed that *ABCA6* was significantly increased in the placenta of obese mothers compared with normal BMI mothers, suggesting that *ABCA6* may participate in abnormal substance transport during obese pregnancy and, therefore, influence offspring development. Moreover, results from the GESA analysis showed that *ABCA6* may participate in oxidative stress (PRNP-PI3K-NOX2 signaling pathway) and inflammation (TLR1/2/4-NFKB signaling pathway) processes, which further underlines the importance of *ABCA6* in maternal obesity and fetal programming.

Nowadays, the publications on *C1orf162* are fairly limited, and the functions related to *C1orf162* protein are largely unknown. In previous bioinformatic studies, *C1orf162* was identified as a signature gene related to co-occurrence of heart failure, lung cancer, polycystic ovary syndrome, and breast cancer (30–32). Here, we found that *C1orf162* may be associated with maternal obesity and fetal programming. Results from our GSEA analysis showed that *C1orf162* actively participates in signaling pathways related to mitochondrial electron transfer in Complex I and inflammatory pathways (TLR1/2/4-NFKB signaling pathway and TNF-NFKB signaling pathway). This evidence, in return, supports the hypothesis that *C1orf162* may be associated with maternal obesity and fetal programming, even though further validation studies are needed in the future.

In this study, the expression of the five signature genes in the overweight data from the same dataset was also explored. The results showed that *C1orf162* was increased while *PTX3*, *HOXB5*, and *ABCA6* exhibited an increased trend in the placenta of overweight mothers compared to mothers with normal BMI. These results, to some extent, supported our findings that *PTX3*, *NCF2*, *HOXB5*, *ABCA6*, and *C1orf162* genes may play a role in maternal obesity and fetal programming. Even though overweight and obesity are two similar conditions and are all associated with excessive fat accumulation, low-grade oxidative stress, and inflammation, obesity is generally considered a more serious condition that initiates a higher pathology. This may explain why the differences observed in obese mothers were more obvious than those with overweight.

In the article where the dataset is from, Cox and colleagues identified several modules (Darkgray, Darkred, and Gray60) that were positively associated with maternal prepregnant BMI and/or newborn birth weight (10). They found that genes in the Darkgray module were positively associated with maternal BMI but were negatively associated with low birth weight in newborn babies. The functions of those co-expression genes from this module were primarily enriched in the defense response to external stimuli. Moreover, Cox et al. found that the Darkred and Gray60 modules were positively correlated with maternal BMI, maternal obesity, and birth weight. Genes from these two modules were involved in processes related to organ and tissue development, blood vessel morphogenesis, and extracellular matrix structure. We noticed that all their findings were on maternal BMI or birth weight but not specifically on the exact categories (Such as overweight, obesity, and low birth weight). In our study, we employed multiple bioinformatic tools focusing on the obese group to identify signature genes related to maternal obesity and fetal programming. Signaling pathway analysis showed that the signature genes identified from our study actively participate in oxidative stress and inflammation in the placenta, which are characteristics of obesity and may affect the placental function and, finally, fetal programming. Moreover, these signature genes also participate in other signaling pathways that are important in nutrition transport in the placenta, cell survival, and cell proliferation. Additionally, we found that one of these signature genes, the *HOXB5*, was negatively associated with newborn birth weight. Therefore, the findings from this study serve as a good complement to the study of Cox et al., which helps to uncover the mechanisms behind maternal obesity and fetal programming.

This study did have its limitations. Firstly, a relatively small sample size in the obese group may lead to a selective bias. Secondly, the findings of this study were based only on bioinformatic analysis of the dataset from a previous study. Even though promising, the functions of these signature genes still require further verification by experimental and molecular studies in the future.

## Conclusion

We identified five signature genes, including *PTX3*, *NCF2*, *HOXB5*, *ABCA6*, and *C1orf162*, which may relate to maternal obesity and fetal programming. Among them, *HOXB5* is implicated directly in newborn birth weight. Moreover, signaling pathways related to these signature genes were listed, which will provide benefits to later researchers.

## Data Availability

The datasets presented in this study can be found in online repositories. The names of the repository/repositories and accession number(s) can be found in the article/Supplementary material.

## Footnotes

1
https://www.ncbi.nlm.nih.gov/geo/
